# Preventive behavior for toxoplasmosis in pregnant adolescents in the state of Ceara, Brazil

**DOI:** 10.1186/1471-2458-12-73

**Published:** 2012-01-24

**Authors:** Fabianne Ferreira Costa, Ana Paula Soares Gondim, Mary Braga de Lima, Jose Ueleres Braga, Luiza Jane Eyre de Souza Vieira, Maria Alix Leite Araújo

**Affiliations:** 1 Master of Collective Health, University of Fortaleza, Av. Washington Soares, 1321, Bairro Edson Queiroz, CEP 60.811-905 Fortaleza, Ceará, Brasil; 2 Internal Medicine Department, State University of Rio de Janeiro, Av. 28 de Setembro, 74 5° Andar, Vila Isabel, CEP 20551030 Rio de Janeiro, RJ, Brasil

## Abstract

**Background:**

When toxoplasmosis is acquired during pregnancy, it can be transmitted to the fetus causing severe lesions in the first two gestational trimesters. This study analyzed the main factors associated with the preventive behavior for toxoplasmosis among pregnant adolescents in the city of Fortaleza in northeast Brazil.

**Methods:**

It is a cross-sectional study conducted from March 2009 to November 2010, with a sample of 320 pregnant adolescents, ages ranging from 12 to 19 years old, receiving prenatal care in the Public Health Care System. Bivariate and multivariate logistic regression model analyses were used to identify the association between preventive behavior for toxoplasmosis, and the independent variables and 95% confidence interval.

**Results:**

We observed that 16.3% of the pregnant adolescents showed preventive behavior for toxoplasmosis. The factors positively associated to the preventive behavior for toxoplasmosis were: age group between 12 and 14 years old (OR = 2.75; 95%CI 1.23-6.12) and more than two prenatal consultations (OR = 2.19; 95%CI 1.17-4.09).

**Conclusions:**

Noteworthy is the importance of a serologic follow-up for pregnant adolescents with clearer and more precise information about risk factors and the importance of adopting preventive behaviors. Thus, it is necessary to establish educational measures for handling food and raising kittens during prenatal care.

## Background

When toxoplasmosis is acquired during pregnancy, it can be transmitted to the fetus causing more severe lesions in the first two gestational trimesters [1,2]. Clinical diagnosis of toxoplasmosis is difficult, considering that circa 90 to 95% of the infected pregnant women do not show any symptoms [3]. Although it results in side effects, the disease has been little studied in terms of its magnitude and importance. In some countries such as Brazil, toxoplasmosis is not a notifiable disease.

In Brazil, according to the technical Manual of the Qualified and Humanized Care for the Prenatal and Puerperal Periods [4], toxoplasmosis screening is recommended in the first prenatal consultation, whenever possible, through the detection of antibodies classified as IgG and IgM. IgG higher than 1:2048 implies the presence of active infection and it should be followed by IgM testing in paired serology. Stable and low IgG antibodies (1:2 to 1:500) can represent previous or persistent infection [5]. The treatment is instituted, for free, in the public health system [4,5]. However, it should be emphasized that even if the service does not provide the test, the health professional is not exempted from providing information about what the disease is, its transmission and preventive care. The main objective of the prenatal and puerperal care is to counsel the woman from the beginning of pregnancy, ensuring, in the end the birth of a healthy child as well as guaranteeing neonatal and maternal well-being [6].

The detection of specific IgM antibodies has been the most used marker for the serologic diagnosis of recent toxoplasmosis. However, the persistence of specific IgM antibodies in some patients and the use of tests with a low specificity have made the interpretation of the serologic results difficult when there is suspicion of toxoplasmosis [7] and may lead to errors in the diagnosis interpretation. The IgM test is considered an unacceptable false positive predictor to evaluate, during pregnancy, the diagnosis of the acute infection of toxoplasmosis [8], while the IgG serologic test contributes to the diagnosis of pregnant women, mainly through serum conversion studies [9].

The serologic diagnosis in pregnant adolescents can be obtained through the following tests: Indirect Immunofluorescence (IIF); Enzyme-Linked Immunoabsorbent Assay (ELISA); Enzyme Linked Fluorescent Assay (ELFA); IgG Avidity Assay and Sabin-Feldman Dye Test (DT); [10,11] Western Blot Reaction [10] and Immunoadsorption Combined with Agglutination (IgM-ISA) [12]. The IIF test can be used in IgG antibodies research [13]. The ELISA is a double antibody sandwich test similar to the IIF for IgM and IgG detection during the first trimester of pregnancy. The ELISA technique is used more because it has a greater sensibility and specificity than the IIF technique [11].

The Avidity Assay is recognized as the linking force between the specific IgG and the epitope of the protein *Toxoplasma gondii*. Besides identifying IgM antibodies in detectable patients, it is the most efficient technique to define the stage of the *T. gondii *infection [7,9]. This test has been used mainly in the acute toxoplasmosis diagnosis of the pregnant adolescent [14], identifying the most probable period of infection, differing the acute infections from the previous ones with only one serum sample [13,15]. Despite helping in the diagnosis of the recent and previous infection by the *T. gondii*, it is necessary to use a serologic tests panel for a more certain infection diagnosis [16].

In the USA, the Centers for Disease Control (CDC) recommend the adoption of individual measures in the primary prevention of toxoplasmosis in pregnancy, based on criteria related to the preventive behavior directed to food hygiene [17].

Most of the studies regarding toxoplasmosis in pregnancy in Brazil [18,19] and other countries [20-28], try to estimate the prevalence of the disease or infection and its determinant factors. As an example, we cite a study with 425 pregnant girls who were receiving prenatal care in basic health care units in the city of Pelotas, Rio Grande do Sul, Brazil which identified that 65% of the pregnant girls did not know about the disease, while most associated it with having cats as pets and less than one third of them associated it with the handling and the consumption of undercooked raw meats and raw vegetables [29]. However, the understanding of the preventive behavior regarding this disease in pregnancy can efficiently contribute to the creation of public policies in Public Health Care System, particularly in the adolescent woman's health area [30,31].

There have been few studies about the preventive behavior in pregnant adolescents. The published works deal with risk behaviors associated to toxoplasmosis in pregnant women [32,33]. Thus, this article analyzes the preventive behavior for toxoplasmosis and its determinants in the Public Health Care System among pregnant adolescents in the state of Ceara, Brazil. Ceara is the third state of the Northeast region of Brazil with 8.439.947 inhabitants, and with 61.9% of this population self-acknowledged as non-white, a Portuguese word for a mix of white, black and indigenous ethnicities. The population of the state is 86.5% catholic and the climate is hot and semi-arid [34].

## Methods

A cross-sectional study was done from March 2009 to October 2010 with pregnant adolescents attended in the prenatal assistance program of the Public Health Care System in the city of Fortaleza, Brazil. Fortaleza is the capital of the state of Ceara, having 2.448.920 inhabitants and an 89.4% rate in relation to the years of formal educational [35]. The Public Health Care System of Fortaleza consists of 92 primary healthcare centers, one medical specialty center, one high-complexity hospital, ten medium-complexity hospitals, and 43 healthcare centers for collection of material for serological tests for toxoplasmosis, divided into six sanitary regions in Fortaleza [34].

For the pregnant adolescents sample size calculations, in the healthcare centers, we used an expected frequency of 50% of pregnant adolescents with preventive behavior for toxoplasmosis, a confidence level of 95% and a margin of error of 5%. Based on these criteria, the minimum expected sample calculated was 341 pregnant adolescents.

Six healthcare centers were selected, one center from each of the six sanitary regions in the city. Two more healthcare centers were included in the divisions which presented a greater number of prenatal consultations.

The pregnant adolescents selection was made through proportionate stratified sampling design. We performed a random poll with the pregnant adolescents registered in each healthcare center of the research to identify the adolescents who were surveyed.

The dependent variable was the preventive and non-preventive behavior for toxoplasmosis. According to the CDC guidelines [17], we considered having a preventive behavior for toxoplasmosis, the pregnant adolescents who matched all the following criteria: not eating undercooked or partially cooked meat weekly, washing fruits and vegetables by immersing them into water with sodium hypochlorite (bleach) before eating them, drinking water from the public water supply, not handling sand, not having kittens as pets, wearing gloves when cleaning the kittens' litter box, cleaning kitchen utensils (cutting boards, dishes, kitchen counters) and washing their hands with warm water and soap after handling raw meat and unwashed fruits and vegetables. The independent variables were: age group, marital status, religion, racial classification, studying, having a paid job, annual family income (US$), number of people living with the pregnant adolescent, first pregnancy, number of living children, gestational age at the first consultation, first consultation of the prenatal assistance, results of the IgG and IgM serology for toxoplasmosis.

The data was collected through a pre-codified and validated questionnaire. The research planning included a pilot study to define the most adequate way to collect the data in the selected services. Undergraduate students in the health care area were trained to identify and interview the study population. The pregnant adolescents was identified through the scheduled list of the initial laboratory tests or while waiting for prenatal assistance. Initially, she was invited to participate in the research and informed about the objectives and methodology of the study. After agreeing with it, the free and informed consent form was signed by the pregnant adolescent when older than 18 or by her parents or legal guardian when she was younger than 18. Then the researcher performed the interview and referred her for the serological test, in which the ELISA technique was used.

The data was stored using the statistical package *Epi Info 3.5.1 *(Centers for Disease Control and Prevention - CDC - Atlanta, Georgia, USA) and analyzed with the statistical package *STATA^® ^*11.0 (Stata Corp LP, College Station, TX 77845, USA). Chi square and Fischer's Exact test were used to analyze the preventive behavior for toxoplasmosis in respect to the independent variables. The differences were considered to be statistically significant with the significance level of 5%. The variable selection for the construction of the multivariate logistic regression model was based on the *Stepwise *process used to identify the possible variables that are predictors of preventive behavior. Adjusted odds ratio (OR) and 95% confidence interval (CI) were calculated by multivariate analysis using logistic regression.

The research was approved by the Ethics and Research Committee of the University of Fortaleza under the No. 052/2008, according to the declaration of Helsinki.

## Results

Of the 341 pregnant adolescents surveyed, 21 were excluded from the study - ten who participated in the pilot study, five who refused to participate, four who were not assisted by their parents or someone responsible for them and two who gave up during the survey. Among the 320 pregnant adolescents who participated in the study only 12% were younger than 15 years of age; 60% were married; 66% declared a religion; racial classification of 76% was non-white, 58% did not study, and 11% had paid jobs. The annual family income varied between US$ 1,026.00 and US$ 102,600.00 and 65% of the families of these pregnant girls received less than US$ 5,436.00. The number of people living with the pregnant adolescent varied from one to 14 people with an average of 3.7 people.

The distribution of pregnant adolescents assisted in the prenatal care in the primary healthcare according to sociodemographic variables of the study is presented in Table 1. The proportion of the pregnant adolescents according to the age group differs statistically regarding the marital status (p = 0.003) and religion (p = 0.010), while racial classification, studying, paid job, annual family income and number of people living with the pregnant adolescent do not differ between the two age groups. The adolescents ranged from 12 to 19 years of age.

**Table 1:** Bivariate analyses of the socio-demographic, biological characteristics and prenatal assistance data related to age group in pregnant adolescents assisted in Public Health Care Services.

Variables	Age group (years)				Total	p
			
	12-14		15-19			
		
	n(%)	95%CI	n(%)	95%CI	n(%)	
**Marital status (n = 320)**						0.003
Single	23(60.5)	44.5-75.0	101(35.8)	30.4-41.5	124(38.7)	
Married	15(39.5)	25.0-55.5	181(64.2)	58.5-69.6	196(61.3)	
**Religion (n = 308)**						0.010
Yes	18(47.4)	32.0-63.1	185(68.5)	59.9-70.9	203(65.9)	
No	20(52.6)	36.9-68.0	85(31.5)	25.0-35.7	105(34.1)	
**Racial classification (n = 320)**						0.109
Non-white	32(84.2)	70.0-93.3	212(75.2)	69.9-79.9	244(76.2)	
White	6(15.8)	6.7-30.0	70(24.8)	20.0-30.1	76(23.8)	
**Studying (n = 320)**						0.075
Yes	21(55.3)	39.4-70.4	113(40.1)	34.5-45.9	134(41.9)	
No	17(44.7)	29.6-60.6	169(59.9)	54.1-65.5	186(58.1)	
**Paid job (n = 320)**						0.073
Yes	1(2.6)	0.1-12.3	35(12.4)	8.9-16.7	36(11.3)	
No	37(97.4)	87.7-99.9	247(87.6)	83.4-91.0	284(88.7)	
**Annual family income (US$) (n = 320)**						0.356
Less than 5436.00	20(52.6)	36.8-68.0	126(44.7)	38.9-50.5	146(45.6)	
More than 5436.00	18(47.4)	31.9-63.1	156(55.3)	49.5-61.0	174(54.4)	
**People living with the pregnant adolescent (n = 320)**						0.306
Less than 4	22(57.9)	41.9-72.7	187(66.3)	60.6-71.7	209(65.3)	
More than 4	16(42.1)	27.3-58.1	95(33.7)	28.4-39.4	111(34.7)	
**First pregnancy (n = 320)**						0.061
Yes	36(94.7)	83.7-99.1	234(83.0)	78.3-87.0	270(84.4)	
No	2(5.3)	0.9-16.3	48(17.0)	12.9-21.8	50(15.6)	
**Living children (n = 50)**						1.000*
1 child	2(100.0)	22.3-0.0	42(87.5)	75.8-94.8	44(88.0)	
2-3 children	0(-)	-	6(12.5)	5.2-24.2	6(12.0)	
**Gestation period in the 1^st ^consultation (n = 315)**						0.220
1^st ^trimester	20(55.6)	39.2-71.0	136(48.8)	42.9-54.6	156(49.5)	
2^nd ^-3^rd ^trimester	16(44.4)	28.9-60.8	143(51.2)	45.4-57.1	159(50.5)	
**First consultation (n = 320)**						0.055
Yes	23(60.5)	44.5-75.0	124(44.0)	38.2-49.8	147(45.9)	
No	15(39.5)	24.9-55.5	158(56.0)	50.2-61.7	173(54.1)	
**Number of prenatal consultations (n = 320)**						0.589
Less than 2	27(71.1)	55.2-83.7	188(66.7)	38.2-49.8	215(67.2)	
More than 2	11(28.9)	16.2-44.7	94(33.3)	28.0-39.0	105(32.8)	
**IgG serology for toxoplasmosis (n = 214)**						0.921
Positive	12(44.4)	26.8-63.2	85(45.4)	38.4-52.6	97(45.3)	
Negative	15(55.6)	36.7-73.2	102(54.6)	47.4-61.6	117(54.7)	
**IgM serology for toxoplasmosis (n = 214)**						0.506*
Positive	0(-)	-	5(2.7)	0.9-6.1	5(2.3)	
Negative	27(100.0)	87.2-0.0	182(97.3)	93.9-99.1	209(97.7)	

Fortaleza, Ceara. March 2009 to November 2010. (n = 320)

*We used Fisher Exact Test

The distribution of pregnant adolescents assisted in the prenatal care in the primary healthcare according to biological characteristics and prenatal assistance variables in the study, is presented in Table 1. The proportion of pregnant adolescents according to age group does not differ statistically regarding the biological characteristics and prenatal care assistance. The distribution of pregnant adolescents according to the first pregnancy shows that 84.4% of them are primigestous. The frequency of living children among the adolescents who already had one child increased between both age groups, being 5% in the age group between 12 and 14 years old and 17% in the age group from 15 to 19 years old. The first trimester of the gestational age to start the prenatal care decreased from 55.6% (95%CI 39.2-71.0) in the age group between 12 and 14 years old to 48.8% (95%CI 42.9-54.6) in the age group from 15 to 19 years old. The first prenatal consultation was more frequent among the pregnant adolescents whose age ranged from 12 to 14 years old, coexisting with the decrease of the number of consultations among the pregnant adolescents with the age from 15 to 19 years old. The number of consultations during prenatal care varied between one and 14 prenatal care consultations and an average of 2.5 consultations. The frequency of positive IgG increased from 44.4% (95%CI 26.8-63.2) in pregnant adolescents from 12 to 14 years old to 45.4% (95%CI 38.4-52.6) in pregnant adolescents from 15 to 19 years old. The presence of positive IgM increased from 0%, in pregnant adolescents from 12 to 14 years old to 2.7% (95%CI 0.9-5.6) in pregnant adolescents from 15 to 19 years old.

Among 307 pregnant adolescents who were asked about preventive behavior for toxoplasmosis, we observed that 16.3% (50) of the pregnant adolescents showed preventive behavior for toxoplasmosis and 83.7% (257) did not show this behavior. Among 50 pregnant adolescents who were asked about preventive behavior for toxoplasmosis, 82.4% did not eat undercooked or partially cooked meat weekly, 15.1% washed fruits and vegetables by immersing them into water with sodium hypochlorite (bleach) before eating them, 92.2% drank water from the public water supply, 93.5% did not handle sand, 78.2% did not have kittens as pets, 25.0% wore gloves when cleaning the kittens' litter box and 9.4% washed kitchen utensils (cutting boards, dishes, kitchen counters) and hands with warm water and soap after handling raw meat, and unwashed fruits and vegetables.

Comparing the preventive behavior for toxoplasmosis among pregnant adolescents, through a bivariate analysis (Table 2), a positive association was verified with the age group between 12 and 14 years (OR = 2.61; 95%CI 1.06-6.04) and two or more prenatal consultations attended by the pregnant adolescents (OR = 2.11; 95%CI 1.09-4.10). No significant associations were found among the pregnant adolescents who were married, followed a religion, non-white racial classification, had an academic history studying, had a paid job, annual family income over US$5436.00, living with less than four people, first pregnancy, gestational age in the first trimester and with positive IgG.

**Table 2:** Bivariate analyses of the factors associated to preventive and non-preventive behavior for toxoplasmosis in pregnant adolescents assisted in Public Health Care Services.

Variables	Behavior				OR(CI95%)	p
	Preventive		Non-preventive			
			
	n(%)	(CI95%)	n(%)	(CI95%)		
**Age group (years) (n = 307)**						
12-14	11(30.6)	17.2-46.9	25(69.4)	53.1-82.7	2.61 (1.06-6.04)	0.013
15-19	39(14.4)	10.6-18.9	232(85.6)	81.0-89.4	1	
**Marital status (n = 307)**						
Married	34(18.3)	13.2-24.3	152(81.7)	75.6-86.7	1.46 (0.74-2.99)	0.241
Single	16(13.2)	8.0-20.1	105(86.8)	79.8-91.9	1	
**Religion (n = 298)**						
Yes	34(17.4)	13.0-23.8	161(82.6)	76.1-86.9	1.14 (0.58-2.36)	0.676
No	16(15.5)	8.7-22.3	87(84.5)	77.6-91.2	1	
**Racial classification (n = 307)**						
No white	37(15.8)	11.5-20.9	197(84.2)	79.0-88.4	1.15 (0.53-2.40)	0.686
White	13(17.8)	10.2-27.8	60(82.2)	72.1-89.7	1	
**Studying (n = 307)**						
Yes	24(18.7)	12.6-26.2	104(81.3)	73.7-87.3	1.35 (0.70-2.61)	0.322
No	26(14.5)	9.9-20.2	153(85.5)	79.7-90.0	1	
**Paid job (n = 307)**						
Yes	6(17.7)	7.4-33.1	28(82.3)	66.8-92.5	1.11(0.36-2.96)	0.819
No	44(16.1)	12.1-20.8	229(83.8)	79.1-87.8	1	
**Annual family income (US$) (n = 307)**						
Less than 5,436.00	24(16.9)	11.4-23.7	118(83.1)	76.3-88.6	0.92 (0.48-1.77)	0.787
More than5,436.00	26(15.8)	10.8-21.9	139(84.2)	78.1-89.2	1	
**People living with the pregnant adolescent (n = 307)**						
Less than 4	35(17.2)	12.4-22.8	169(82.8)	77.2-87.5	1.21 (0.61-2.53)	0.561
More than 4	15(14.6)	8.7-22.3	88(85.4)	77.6-91.2	1	
**First pregnancy (n = 307)**						
Yes	42(16.1)	12.0-21.0	218(83.9)	79.0-87.9	1.06 (0.40-2.52)	0.882
No	8(17.0)	8.2-29.7	39(83.0)	70.2-91.7	1	
**Gestation period in the 1^st ^consultation (n = 303)**						
1^st ^trimester	28(18.7)	13.0-25.5	122(81.3)	74.5-86.9	1.37 (0.71-2.65)	0.314
2^nd^-3^rd ^trimester	22(14.4)	9.4-20.6	131(85.6)	79.3-90.5	1	
**Number of prenatal consultations (n = 307)**						
More than 2	24(23.5)	16.0-32.4	78(76.5)	67.5-83.9	2.11 (1.09-4.10)	0.015
Less than 2	26(12.7)	8.6-17.7	179(87.3)	82.2-91.3	1	
**IgG serology for toxoplasmosis (n = 208)**						
Positive	20(21.3)	13.8-30.4	74(78.7)	69.6-86.1	1.78 (0.80-4.00)	0.119
Negative	15(13.2)	7.8-20.3	99(86.8)	79.6-92.1	1	

Fortaleza, Ceara. March 2009 to November 2010. (n = 307)**

**We did not have information about prevent behavior for 13 patients

After adjusting the model, the factors associated with a higher chance of occurrence of preventive behavior with statistical significance was the age group between 12 and 14 years old (OR = 2.75; 95%CI 1.23-6.12) and two or more prenatal consultations (OR = 2.19; 95%CI 1.17-4.09) (Table 3).

**Table 3:** Multivariate logistic regression analyses of factors associated to the preventive behavior for toxoplasmosis in pregnant adolescents assisted in Public Health Care Services.

Variables	OR (CI95%)	p
**Age group (years)**		
12-14	2.75 (1.23-6.12)	0.013
15-19	1	
**Number prenatal consultations**		
More than 2	2.19 (1.17-4.09)	0.014
Less than 2	1	

Fortaleza, Ceara. March 2009 to November 2010. (n = 307)**

**We did not have information about prevent behavior for 13 patients

## Discussion

The factors positively associated to the preventive behavior for toxoplasmosis was the age group between 12 and 14 years old and two or more prenatal consultations. The preventive behavior may show a strong relation with eating habits, such as washing fruits and vegetables by immersing them into water with sodium hypochlorite (bleach) before eating them and cleaning kitchen utensils (cutting boards, dishes, kitchen counters), washing hands with warm water and soap after handling raw meat and unwashed fruits and vegetables and also having direct contact with kittens [36,37]. The data of our study and its cross-sectional methodological design are not enough to conclude what caused this association.

Unfortunately, we are not able to identify the hotspot of toxoplasmosis occurrence, because we did not study the patients from all the sanitary regions of Fortaleza. Indeed we included eight health services and four sanitary regions from the city.

The pregnant adolescents eat meat an average of 2.3 times a week. The preventive behavior for toxoplasmosis of the pregnant adolescents can be associated to eating changes that occur during pregnancy, involving subjective values essential to adolescence and pregnancy [38].

The behaviors of the pregnant adolescents to prevent toxoplasmosis in pregnancy are often associated to behavioral factors, such as, consumption of fresh and undercooked meat and raw vegetables (17.6%) and having kittens as pets (21.8%). This study was dissimilar to the findings of another study [18], which showed the consumption of these foods by 58.2% of the 127 pregnant girls who were studied. The contact with kittens is a factor associated with toxoplasmosis in pregnant adolescents [39,40].

In Northeast Brazil, particularly in the state of Ceara, two studies conducted with pregnant girls identified the consumption of homemade frozen sugar water in Brazil, known as "dindin" [41,42], implying that the contamination of the water used in the homemade preparation by oocysts is the most probable infection source. Thus, it is emphasized the importance of adequate treatment of water for human consumption, mainly in places where the water supply is not treated. The information about the correct freezing and boiling of water must be given to the pregnant women facing these situations in order to prevent a possible infection.

The adequate cleaning of kitchen utensils and washing of hands after having contact with raw meat and unwashed fruits and vegetables presented a low frequency, resulting in a higher risk of acquiring the *T. gondii*.

The prevalence (45.3%) of anti-*Toxoplasma gondii *IgG antibodies observed in this study is similar to the results of positive IgG found in pregnant adolescents from some regions of Brazil, such as, Belo Horizonte, Minas Gerais; [43] Araraquara, state of São Paulo; [44] and Londrina, state of Paraná [45]. This finding differs from other studies done in Fortaleza, Ceara, which has a high prevalence of previous infections in pregnant adolescents (91.7%, from 12 to 15 years old and 63.5% from 16 to 18 years old) probably because Susan Sroka [42], the researcher, studied a sample of a maternity of reference in the state of Ceara. Our study group could be considered a primary health care population and not a hospital based group [42]. Thus, we must consider public health care programs that contemplate a systematic screening of pregnant adolescents and neonates for prevention, diagnosis and early treatment of the affected neonates, generally asymptomatic at birth and also stimulate preventive behaviors by the pregnant adolescent during prenatal care [46].

In Brazil, the serologic triage for toxoplasmosis is recommended in the first prenatal consultation, since it is available in the health care service, what is not usually performed in the primary health care, although the pregnant adolescents under 15 years old must be considered [47], according to the Ministry of Health guidelines, as a risky pregnancy. Considering this, they are directed to specialized services and some of them establish assistential guidelines for toxoplasmosis from the detection to the treatment [47].

The occurrence of five IgG and IgM seropositivity pregnant adolescents was similar to the results found in another study. As a new serology and IgG Avidity Assay Tests were not performed, the interpretation of IgM results had to be cautious in order to avoid the exposure of the mother and fetus to unnecessary procedures [48], showing the importance of the diagnostic confirmation. The Avidity Assay was not performed because this study does not perform serum prevalence analysis.

The importance of a serologic follow-up for the pregnant adolescents with clearer and precise information about the risk factors and the importance of adopting preventive behaviors, is noteworthy. Thus, it is necessary to establish more global preventive measures considering social, economic and cultural matters.

The high percentage of pregnant adolescents with non-preventive behavior stands out, which shows the lack of information about preventive care. We add to this the greater proportion of low family income and little formal schooling among the pregnant adolescents observed in the study that can contribute to increase the risk for infection and contamination of the fetus. It has been estimated that the number of pregnant adolescents in specialized hospitals is increasing.

Moreover, it is understood that the information regarding preventive measures for toxoplasmosis are not exclusive to health professionals. But include a set of actions developed by global public policies of health and education, along with the professional training on the study theme.

## Conclusions

We concluded that there is a strong association between younger pregnant adolescents who had more prenatal consultations and the preventive behavior for toxoplasmosis in pregnant adolescents assisted in primary health care in the city of Fortaleza, Ceara, Brazil. The importance of a serologic follow-up for the pregnant adolescents with clearer and precise information about the risk factors and the importance of adopting preventive behaviors, is noteworthy. Thus, it is necessary to establish educational measures for food-handling and raising kittens during prenatal care although more global preventive measures can be implemented, considering social, economic and cultural matters.

Moreover, it is understood that the information about preventive measures for toxoplasmosis are not exclusive to health professionals, but include a number of actions with the inclusion of this appeal to the list of compulsory notifications, together with the health professional training on the study theme.

## Competing interests

We recognize that this manuscript does not present financial conflicts among the authors. We certify that this manuscript was submitted only and exclusivity to the BMC Public Health.

## Authors' contributions

Gondim APS, Costa FF and Lima MB conceived and supervised analyses of the study. Vieira LJES, and Braga JU, completed the analyses. Araújo MAL assisted with the study and analyses. All authors read and approved the final manuscript.

## Authors' information

Fabianne Ferreira Costa, Ms, University of Fortaleza

Ana Paula Soares Gondim, PhD, Professor, University of Fortaleza

Mary Braga de Lima, Ms, Professor, University of Fortaleza

Jose Ueleres Braga, PhD, Professor, State University of Rio de Janeiro

Luiza Jane Eyre de Souza Vieira, PhD, Professor, University of Fortaleza

Maria Alix Leite Araújo, PhD, Professor, University of Fortaleza

## Pre-publication history

The pre-publication history for this paper can be accessed here:

http://www.biomedcentral.com/1471-2458/12/73/prepub
